# Genetic variants of MARCO are associated with susceptibility to pulmonary tuberculosis in a Gambian population

**DOI:** 10.1186/1471-2350-14-47

**Published:** 2013-04-23

**Authors:** Dawn ME Bowdish, Kaori Sakamoto, Nathan A Lack, Philip C Hill, Giorgio Sirugo, Melanie J Newport, Siamon Gordon, Adrian VS Hill, Fredrick O Vannberg

**Affiliations:** 1 McMaster Immunology Research Centre, McMaster University, Hamilton, ON, L8S 4K1, Canada; 2 Department of Pathology, College of Veterinary Medicine, University of Georgia, 501 D.W. Brooks Drive, Athens, GA, 30605, USA; 3 School of Medicine, Koc University, Istanbul, 34450, Turkey; 4 Medical Research Council Laboratories, Fajara, The Gambia; 5 Centre for International Health, Department of Preventive and Social Medicine, University of Otago, School of Medicine, Dunedin, New Zealand; 6 Ospedale San Pietro Fatebenefratelli, Rome, Italy; 7 Medical School Research Building, Brighton & Sussex Medical School, Brighton, East Sussex, BN1 9PS, UK; 8 Sir William Dunn School of Pathology, University of Oxford, 10 South Parks Road, Oxford, OX1 3RE, UK; 9 The Wellcome Trust Centre for Human Genetics, Roosevelt Drive, Oxford, OX3 7BN, UK; 10 School of Biology, Georgia Institute of Technology, 310 Ferst Drive, Atlanta, GA, 30332, USA

**Keywords:** Scavenger receptors, *Mycobacterium tuberculosis*, Single nucleotide polymorphisms, Case control study, MARCO

## Abstract

**Background:**

The two major class A scavenger receptors are scavenger receptor A (SRA), which is constitutively expressed on most macrophage populations, and macrophage receptor with collagenous structure (MARCO), which is constitutively expressed on a more restricted subset of macrophages, (e.g. alveolar macrophages) but whose expression increases on most macrophages during the course of infection. Although the primary role of SRA appears to be clearance of modified host proteins and lipids, mice defective in expression of either MARCO or SRA are immunocompromised in multiple models of infection and *in vitro* assays, the scavenger receptors have been demonstrated to bind bacteria and to enhance pro-inflammatory signalling to many bacterial lung pathogens; however their importance in *Mycobacterium tuberculosis* infection, is less clear.

**Methods:**

To determine whether polymorphisms in either SRA or MARCO were associated with tuberculosis, a case–control study of was performed. DNA samples from newly-detected, smear-positive, pulmonary tuberculosis cases were collected from The Gambia. Controls for this study consisted of DNA from cord bloods obtained from routine births at local Gambian health clinics. Informed written consent was obtained from patients or their parents or guardians. Ethical approval was provided by the joint The Gambian Government/MRC Joint Ethics Committee.

**Results:**

We studied the frequencies of 25 polymorphisms of *MSR1* (SRA) and 22 in *MARCO* in individuals with tuberculosis (n=1284) and matched controls (n=1349). No SNPs within the gene encoding or within 1 kb of the promoter sequence of MSR1 were associated with either susceptibility or resistance to tuberculosis. Three SNPs in *MARCO* (rs4491733, Mantel-Haenszel 2x2 *χ*2 = 6.5, *p* = 0.001, rs12998782, Mantel-Haenszel 2x2 *χ*2 = 6.59, *p* = 0.001, rs13389814 Mantel-Haenszel 2x2 *χ*2 = 6.9, *p* = 0.0009) were associated with susceptibility to tuberculosis and one (rs7559955, Mantel-Haenszel 2x2 *χ*2 = 6.9, *p* = 0.0009) was associated with resistance to tuberculosis.

**Conclusions:**

These findings identify MARCO as a potentially important receptor in the host response to tuberculosis.

## Background

*Mycobacterium tuberculosis*, the causative agent of tuberculosis, is one of few pathogens that infects and persists in host macrophages. Consequently it has successfully colonized up to a third of the world’s population and of those, many millions develop active disease [1]. Phagocytosis of *M. tuberculosis* is mediated by a number of receptors including the mannose receptor and DC-SIGN, which recognise mannose-capped lipoarabinomannan (Man-lam) [2,3], complement receptor via recognition of opsonin-coated bacteria [4], and others [5]. Although the class A scavenger receptors, SRA (class A scavenger receptor) and MARCO (macrophage receptor with collagenous structure), are broadly classified as “phagocytic receptors”, and have been demonstrated to internalize mycobacterial species such as *M. leprae*[6], *M. avium*[7], *M. bovis* Bacille Calmette-Guérin [8,9], and *M. tuberculosi*s [10,11], it has been proposed that there is functional redundancy in mycobacterial uptake by macrophages so that any individual receptor is dispensable [10,12]. Intriguingly, whereas uptake of *M. tuberculosis* into alveolar macrophages during the course of acute infection may be mediated by many equivalent receptors, long-term extra-pulmonary persistence (e.g. in the adipose tissue), may be mediated through scavenger receptor uptake [13].

Although the importance of phagocytic receptors in uptake of *M. tuberculosis* is not entirely clear, their role in induction of pro-inflammatory responses appears to be more straightforward. Class A and B scavenger receptors are required for maximal cytokine responses to mycobacterial lipoarabinomannans [14] and lipopeptides [15], and both the class A scavenger receptors and the C-type lectin, Mincle, are required for optimal toll-like receptor (TLR) and Syk/Card9 signaling responses to mycobacterial trehalose dimycolate (cord factor), respectively [16,17]. The biological importance of this enhancement of cytokine responses remains to be fully elucidated; however, in lung infection models of *M. tuberculosis,* the absence of SRA is protective [18], but in models of disseminated disease, its absence is fatal [8,11,19].

To some degree, the lack of clarity surrounding whether these receptors are of key importance in host defence towards tuberculosis is probably due to deficiencies in the mouse model. The lung pathology of tuberculosis is sufficiently different in mice, in that many of the hallmark features of disease (e.g. granulomas) do not occur, and genes found to be associated in human studies are not necessarily associated with murine susceptibility and *vice versa* (reviewed in [20]). In order to determine whether the class A scavenger receptors are crucial to protection against human disease, we have performed a case–control study of single nucleotide polymorphisms (SNPs) using samples from a well-described Gambian population [21,22]. We demonstrate that polymorphisms in *MARCO,* but not *MSR1,* the gene encoding SRA, are associated with tuberculosis infection in the Gambian population. Encouragingly, these results are consistent with a recently published case–control study in a Chinese Han population (n= 923 cases and 1033 controls) [23]. In both the Chinese Han and Gambian populations, SNPs in intron 1, which we identify as a putative alternative promoter site, are associated with susceptibility to tuberculosis, implying that changes in MARCO function or expression contribute to host defence against tuberculosis.

## Methods

### Patient DNA Samples

DNA samples from newly-detected, smear-positive, pulmonary tuberculosis cases and healthy controls were collected from The Gambia as described [21,22]. Samples were comprised of cases and controls (please see Table 1 for details). Gambian pulmonary tuberculosis cases (n=1,498) presented with a compatible clinical picture of tuberculosis and were diagnosed with culture or smear positivity; all cases that were smear positive but culture negative had radiographic confirmation. Exclusion criteria included presentation of autoimmune, cancer, or other diseases, such as HIV-1, which are known to impact host immunity. The majority (>95%) of the tuberculosis cohort was screened for HIV-1, with positive cases excluded from the study because HIV infection increases the risk of tuberculosis. Gambian controls (n=1,496) were recruited from routine births at local Gambian health clinics. Some samples were removed from the study due to quality control issues, including low genotype frequency and relatedness, leaving 1284 cases and 1349 controls for the final analysis. These cases and controls have been previously analyzed studying tuberculosis (46) and malaria [24], and we have a detailed understanding of the underlying ethnic stratification through genome-wide SNP data. Logistic regression based upon 6-axis of variation (to reduce the impact of ethnicity on the final association analysis) did not affect our final conclusions. Informed written consent was obtained from patients or their parents or guardians. Ethical approval was provided by the joint The Gambian Government/MRC Joint Ethics Committee.

**Table 1:** Characteristics of study population

	**Status**	**Control**		**Case**		**Total**	P value*
**Gender**	*Male*	712	40.6%	1043	59.4%	1755	<0.001
	*Female*	784	63.3%	455	36.7%	1239	
**Ethnicity**	*Mandinka*	454	47.8%	495	52.2%	949	0.250
	*Jola*	287	56.9%	217	43.1%	504	0.004
	*Fula*	296	58.7%	208	41.3%	504	<0.001
	*Wollof*	263	71.3%	106	28.7%	369	<0.001
	*Other*	190	28.8%	470	71.2%	660	<0.001
	*Unknown*	6	75.0%	2	25.0%	8	
	*Total*	1496		1498		2994	

*Standard Chi-Square tests with 1 degree of freedom were performed for comparing groups.

### Genotyping

Polymorphisms in *MSR1* and *MARCO* genes were identified from the National Center for Biotechnology Information dbSNP database (http://www.ncbi.nlm.nih.gov/projects/SNP/). SNPs were genotyped using the Sequenom (San Diego) MassARRAY system, and the primer extension products were analyzed using MALDI-TOF mass spectrometry [25,26].

### Bioinformatic analysis

Potential alternative splice sites of MARCO were investigated using the Alternative Splice Site Predictor (ASSP) tool (http://wangcomputing.com/assp/index.html) [27]. Alignments of intron 1 and regions of high homology between multiple species were determined using ESPERR (evolutionary and sequence pattern extraction through reduced representations) [28]. To investigate whether there might be conserved transcriptional elements in intron 1 of the gene encoding MARCO, the Transcription Element Search System (TESS) program was used (http://www.cbil.upenn.edu/tess) [29].

### Statistical analysis

**Figure 1:**
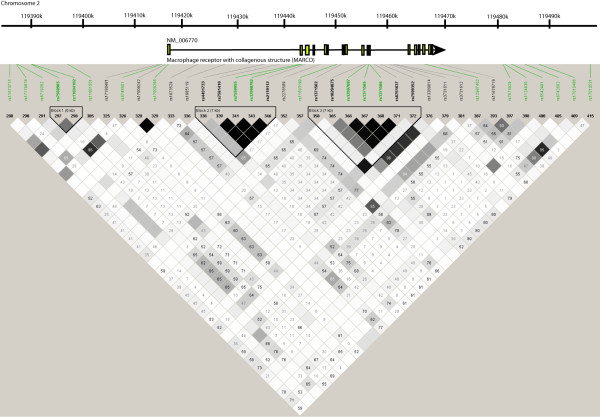
**Haploview analysis of SNPs in *MARCO *[GeneID: 8685], located on chromosome 2q12.** Linkage Disequilibrium (LD) in the African population is visualized across the *MARCO* locus, including upstream and downstream regions (chr2: 119414167–119468337). Polymorphisms are identified by their dbSNP rs numbers, and their position relative to the gene structure is marked. SNPs marked in bold were typed in this study. SNPs marked in green were included in the Wellcome Trust Case Control consortium [32] analysis and were used in validation studies. *Empty squares* indicate a high degree of LD (LD coefficient D' = 1) between pairs of markers. Numbers indicate the D' value expressed as a percentile. *Black squares* indicate pairs in strong LD with logarithm of odds (LOD) scores for LD ≥2; *grey squares*, D' < 1 with LOD ≥2; *white squares*, D' < 1.0 and LOD < 2.

Statistical analysis of genotype associations was performed using SPSS version 12.0 (SPSS, Inc., Chicago, IL). Analysis of linkage disequilibrium (LD) and haplotypes (Figure 1) was performed using the Haploview version 3.2 program [30]. Haplotype blocks were defined as regions demonstrating strong evidence of historical recombination between less than 5% of single-nucleotide polymorphism (SNP)–pair comparisons [31]. All control genotype distributions were in Hardy-Weinberg equilibrium (0.05 level).

## Results

### SNPs in MARCO are associated with resistance/susceptibility to tuberculosis

We chose 25 tag SNPs in *MARCO* and 22 in *MSR1*; preliminary analysis indicated that these SNPs were polymorphic and found at reasonable frequencies in the Gambian population (e.g. > 5%). None of the SNPs genotyped in *MSR1* were associated with tuberculosis susceptibility (Additional file 1 Table S1). Three SNPs in *MARCO* showed some evidence of association with susceptibility to tuberculosis (*p* < 0.02, Table 2), and for one of these, rs7559955, heterozygotes showed reduced risk of tuberculosis (*p* = 0.008, Table 3). These SNPs were located within two regions of the *MARCO* gene. The first cluster of three SNPs is located in intron 1 of the gene (rs4491733, rs7559955, rs12998782), and 1 SNP (rs13389814) was found in intron 16.

**Table 2:** **Association analysis of *MARCO *SNPs in a Gambian tuberculosis case control study**

**CHR**		**SNP**	**Position**	**Genotypic**		**Dominant**	
2		rs17180481	119414167	5.2	7.60E-02	4.2	4.10E-02
2		rs6748401	119414287	3.9	1.40E-01	0	8.30E-01
2		rs6748401	119414527	2.6	2.80E-01	0.5	4.90E-01
2		rs17009242	119415253	1.6	4.50E-01	1.6	2.10E-01
2		rs17009268	119415479	NA	NA	NA	NA
2		rs1073529	119421449	1.8	4.20E-01	1.7	1.90E-01
2		rs1985119	119426870	1.3	5.20E-01	1.2	2.70E-01
2		rs4491733	119434498	12.3	2.10E-03	6.5	1.10E-02
2		rs7559955	119441199	**7.8**	**2.00E-02**	**6.9**	**8.50E-03**
2		rs12998782	119441610	7.3	2.60E-02	6.5	1.10E-02
2		rs2119112	119442389	1	6.10E-01	0.4	5.10E-01
2		rs2278589	119445346	0	9.90E-01	0	9.60E-01
2		rs11693199	119448272	5.9	5.10E-02	3.1	7.90E-02
2		rs1371562	119449418	0.1	9.30E-01	0.1	7.10E-01
2		rs10204875	119453881	2.9	2.40E-01	1.4	2.30E-01
2		rs12997087	119454047	0.6	7.60E-01	0.4	5.20E-01
2		rs1371565	119454355	0.5	7.80E-01	0.5	4.90E-01
2		rs1371566	119454417	0.5	7.60E-01	0.4	5.50E-01
2		rs6761637	119455533	1.1	5.80E-01	1	3.20E-01
2		rs7599352	119456760	1.5	4.70E-01	1.1	2.90E-01
2		rs13389814	119458420	**8**	**1.90E-02**	**6.9**	**8.50E-03**
2		rs3731611	119459081	1.7	4.30E-01	1.6	2.10E-01
2		rs3731612	119459335	1.7	4.30E-01	1.6	2.10E-01
2		rs12987402	119465026	1.6	4.40E-01	1.3	2.50E-01
2		rs11678719	119468337	5.1	7.70E-02	0.7	4.00E-01

**Table 3:** The T allele of rs7559955 shows heterozygote protection

	**Control**	**Tuberculosis**	**Total**
**C/C**	**542**	**581**	1123
**C/T**	**651**	**553**	1204
**T/T**	**156**	**150**	306
**total**	1349	1284	2633
	40.2%	45.2%	
	48.3%	43.1%	
	11.6%	11.7%	
HW P	0.060	0.295	

### The T variant in rs7559955 may confer a regulatory element

**Figure 2:**
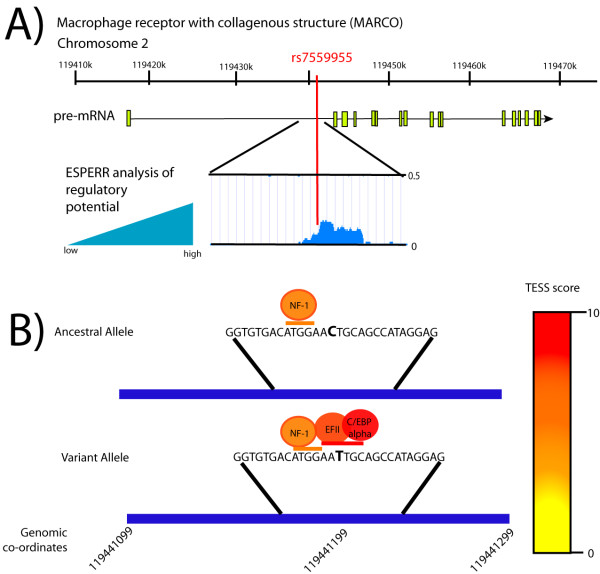
**TESS Analysis of potential transcription factor binding.** The T variant allele creates potential for EFII and C/EBPalpha binding. **A**) The rs7559955 allele in intron 1 is found in a region of high conservation. Alignments of the homologous region from human, chimpanzee, macaque, mouse, rat, dog, and cow were performed, and the region of high conservation, and thus high regulatory potential as determined by ESPERR, is marked in blue. **B)** Transcription factor binding site analysis using TESS indicates that the presence of the T allele may confer the addition of EFII or C/EBPalpha transcription factor binding sites.

The presence of a polymorphism in rs7559955 is associated with resistance to tuberculosis and may confer heterozygote protection (Table 3). Bioinformatic analysis using ESPERR indicates that the 200 bp region surrounding rs7559955 contains a relatively short sequence that is highly conserved amongst seven species (human, chimpanzee, macaque, mouse, rat, dog, and cow) and is thus a potential regulatory sequence (Figure 2A). Score values above the 0.1 default upper limit indicate very marked resemblance to alignment patterns typical of regulatory elements [33,34]. We thus hypothesized that this region might be a transcription factor binding site. Analysis using Transcription Element Search System (TESS) indicated that there was a weak match in both the ancestral and variant allele for the transcription factor NF-1 (score 8.0). The T allele appears to result in the addition of a transcription factor binding site that has homology to that of EFII (score 9.155) or C/EBPalpha(score 10) [29] (Figure 2B).

## Discussion

Herein, we demonstrate that polymorphisms within the human class A scavenger receptor MARCO correlate with susceptibility/resistance to tuberculosis in a Gambian population. Interestingly, none of the SNPs identified in this study encoded non-synonymous mutations, but rather were within introns (i.e. rs13389814 within intron 16) and putative regulatory regions (i.e. rs7559955 within a potential promoter site in intron 1). This may imply that changes in MARCO expression rather than overt changes in structure are key to conferring resistance or susceptibility to tuberculosis. In fact, although SRA (encoded by *MSR1*) shares high amino acid similarity with MARCO (76% amino acid similarity) and has many overlapping ligands, the two receptors differ considerably at the level of regulation of expression. Whereas SRA expression is regulated primarily by factors associated with macrophage differentiation (e.g. GM-CSF) and lipid accumulation, MARCO is primarily regulated by inflammation and bacterial infection (reviewed in [35]). Unlike SRA, which is expressed on virtually all macrophages, MARCO is constitutively expressed on some subsets of macrophages (i.e. alveolar, peritoneal, marginal zone of the spleen, and medullary cords of the lymph nodes), and is rapidly up-regulated on others during the course of infection. Indeed, *in vivo* MARCO expression is quickly (<1 h) increased upon challenge with bacteria or bacterial products, and this increase in expression occurs even at sites distal to bacterial challenge (e.g. on Kupffer cells post lung infection) [36,37]. Changes in MARCO expression during the course of experimental tuberculosis infections have not been performed, but expression of MARCO increases upon systemic challenge with BCG [37] and is higher on macrophages within and proximal to BCG-containing granulomas. MARCO-expressing macrophages in the marginal zone appear to phagocytose more BCG than neighbouring macrophages that do not express MARCO [9]. Whether the presence of a SNP within intron 1 (rs7559955) results in enhanced or decreased expression of *MARCO,* constitutively or after exposure to pathogens or their products, and whether changes in expression alter binding, uptake, or cytokine production, requires further investigation.

Recently Ma *et al.* reported that SNPs in *MARCO* were associated with susceptibility to tuberculosis in the Chinese Han population [23]. Interestingly, they identified that rs17009716 (chromosomal position 119441930), was significantly associated with susceptibility to tuberculosis. This SNP is also within intron 1, less than 400 base pairs from and in high LD with rs12998782 and rs7559955 in Chinese (CHB), European (CEU), and African (YRI) populations. This provides support for potential consistency of a MARCO association across diverse populations and suggests a role for MARCO, in host defence against tuberculosis, possibly at the level of gene regulation, although further functional studies are warranted. Although animal models using MARCO knockout mice strongly associate MARCO with many pulmonary infections, such as pneumonia and influenza [38-41], whether polymorphisms in MARCO are associated with susceptibility to infection in humans remains to be determined. A recent Danish study found that non-synonymous SNPs within MARCO were found at very low frequency (0.005-5%) within a Danish population, and in a study investigating whether MARCO was associated with chronic obstructive pulmonary disease (COPD) and lung infection in COPD patients, only one of these was associated with an increased risk of sepsis and none with pneumonia or COPD itself [42]. Unfortunately, the intronic SNPs identified in our study and in the Chinese Han population [23] were not investigated. It will be essential to validate these studies in more diverse populations, and perhaps to investigate more SNPs, in order to confirm the relevance of these results.

The observed association of MARCO variants with sepsis is consistent with *in vivo*[36,37,43,44] and *in vitro*[44,45] observations that MARCO regulates pro-inflammatory cytokine production to whole bacteria and TLR ligands. The class A scavenger receptors in general, and MARCO in particular, have been demonstrated to enhance TLR signalling to mycobacterial cell wall components, such as the major immunogenic lipid on the mycobacterial cell wall, trehalose dimycolate (TDM) [46]. Whether this increased regulation of cytokines is of benefit or is detrimental to the host during the course of either pulmonary or systemic tuberculosis infection remains to be determined.

## Conclusions

In summary, we have demonstrated that genetic variation in a class A scavenger receptor, MARCO, is associated with susceptibility/resistance to tuberculosis in a Gambian population, consistent with previously reported data from a Chinese Han population [23]. Further studies are warranted to determine whether genetic variation in MARCO may alter expression of the receptor, and whether regulation of MARCO expression contributes to protection or susceptibility to tuberculosis at the level of macrophage infection, initiation of a pro-inflammatory response, or long-term persistence.

## Abbreviations

SR: Scavenger receptor; SNP: Single nucleotide polymorphism; TLR: Toll-like receptor; MARCO: Macrophage receptor with collagenous structure; WTCCC: Wellcome Trust Case Control Consortium; SRCR: Scavenger receptor cysteine-rich domain; PBMC: Peripheral blood mononuclear cell

## Competing interests

The authors have no competing interests.

## Authors’ contributions

DMEB, KS, NL and FOV performed experiments. PCH, GS, MN, AVSH provided infrastructure and support for sample collection and processing. DMEB, SG, AVSH, FOV designed the experiments. FOV performed the statistical analysis. KS, SG, and AVSH provided funding. All authors read, edited, commented and approved on the manuscript.

## Pre-publication history

The pre-publication history for this paper can be accessed here:

http://www.biomedcentral.com/1471-2350/14/47/prepub

## Supplementary Material

Additional file 1: Table S1 Genotyped SNPs in *MSR1.* Click here for file
