# Supplementary material for: Genetic variants of MARCO are associated with susceptibility to pulmonary tuberculosis in a Gambian population
Source: BMC Med Genet. 2013 Apr 23;14:47. doi: 10.1186/1471-2350-14-47 (PMC3652798; doi:10.1186/1471-2350-14-47)
Supplement: Additional file 1: Table S1 — Genotyped SNPs in MSR1. [file 1471-2350-14-47-S1.pdf]

Supplemental Table 1. Genotyped SNPs in *MSR1*.

SNP	CHR	POSITION
rs13262326	8	15958060
rs12678046	8	15970070
rs1904577	8	15971684
rs17620619	8	15981722
rs17677251	8	15985518
rs2170115	8	15987187
rs12718376	8	15998145
rs351556	8	16004010
rs1564819	8	16005211
rs351554	8	16007395
rs3747531	8	16012648
rs13306543	8	16021585
rs13306549	8	16026284
rs1531652	8	16027195
rs11988710	8	16033108
rs398095	8	16041765
rs394447	8	16048146
rs364558	8	16050479
rs3827509	8	16051652
rs3810780	8	16051850
rs3827510	8	16052087
rs7815674	8	16059926
rs351581	8	16053426
rs6530946	8	16054928
